# The narcissistic personalities of mothers as perceived by their daughters and its relationship to emotional balance among female students at King Faisal University

**DOI:** 10.3389/fpsyg.2025.1629470

**Published:** 2025-07-17

**Authors:** Entesar Alnashmi, Hanem M. Alboray

**Affiliations:** Department of Education and Psychology, College of Education, King Faisal University, Al-Ahsa, Saudi Arabia

**Keywords:** narcissism, narcissistic mother, emotional balance, personality traits, female university students

## Abstract

**Introduction:**

This study explores how female university students perceive their mothers’ narcissistic traits and investigates the relationship between these perceptions and the students’ emotional balance.

**Methods:**

A descriptive correlational design was used. The sample included 416 female students aged 18 -24 from King Faisal University. Two tools were utilized: the Narcissistic Mother Scale and the Emotional Balance Scale developed by the researcher.

**Results:**

Overall, students perceived their mothers’ narcissism as low, except for the “excitability” dimension, which scored moderate. Emotional balance levels among students were also moderate. A significant negative correlation was found between perceived maternal narcissism and emotional balance. “Intolerance” was the strongest predictor of emotional imbalance, followed by “exploitative Ness.”

**Discussion:**

These findings suggest that higher levels of perceived maternal narcissism may negatively influence daughters’ emotional balance, highlighting the potential impact of maternal personality traits on young women’s emotional development.

## Introduction

Personality traits significantly influence an individual’s ability to adapt to life. While personality is typically considered normal, any imbalance can manifest as complex and maladaptive behavioral patterns, including narcissistic personality (Abu Shandi, 2014).

Parental narcissism, particularly maternal narcissism, poses a substantial challenge within the family dynamic. Narcissistic parents often struggle to express emotions toward their children, potentially leading to disorganized attachment and impulsive behaviors in offspring (Livesley, 2007). Studies have revealed a strong positive correlation between maternal narcissism and daughters’ narcissism, whereas maternal narcissism was negatively associated with daughters’ expressions of empathy (Dutton et al., 2011). A salient characteristic of narcissistic mothers in their relationship with daughters is a lack of empathy, which can foster feelings of insignificance in the daughters. In many cases, daughters suppress their emotions to avoid harsh criticism from their mothers, having learned early that external appearances outweigh emotions and that emotional suppression is a means to avoid pain (Liebrecht, 2021).

Emotional balance is characterized by stability, calmness, and self-regulation in emotional and behavioral responses, alongside impulse control (Al-Safi, 2018; Gross, 2015, p. 12). Conversely, instability reflects an imbalance in this equilibrium and mood fluctuations (Diagnostic and Statistical Manual of Mental Disorders, Fifth Edition, 2013). An emotionally balanced individual possesses the skills to identify stressors and develop healthy coping mechanisms to maintain their emotional equilibrium. In contrast, an emotionally imbalanced person lacks these skills, rendering them more susceptible to mood swings, aggressive reactions, and harmful behaviors when confronted with stress (Bhagate et al., 2015). Consequently, emotional traits are among the most crucial factors influencing an individual’s social and psychological adjustment.

Given the critical importance of the university stage as a transitional phase demanding emotional balance for academic and social success and considering the pivotal role of the mother in her children’s – especially daughters’ – psychosocial and cognitive development, this study represents a crucial step toward understanding how university students’ perceptions of their mothers’ narcissistic personality traits can predict their emotional balance. This research aims to enrich the Arabic literature with scientific content elucidating the relationship between perceived maternal narcissism and emotional balance among female university students in Saudi society, a topic where studies remain scarce. Furthermore, its findings may help identify factors influencing students’ emotional balance, provide recommendations for enhancing their mental health, and increase mothers’ awareness of their fundamental role in fostering healthy development.

## Theoretical framework

### Narcissistic personality

The study of personality has received widespread attention from psychologists seeking to deconstruct its constituent elements, recognizing them as the basic construction of cognitive-emotional functioning and the basic basis for understanding psychological phenomena, personality is defined as “a set of behavioral and mental traits characterized by stability and forming individual differences among humans within a specific culture. Therefore, personality disorders are defined based on the culture, expectations, and customs of society” (Ali, 2018, p. 95). The president of the Psychometric Society, Guilford (1942) believed that personality contains types of traits that he considered components of a relatively stable general style that differs from one individual to another. These characteristics include physiological, behavioral, mental, and temperamental traits (Akhrass, 2012).

Traits are defined as “basic units in the organization of personality that we cannot see but infer their existence through behavior. It is rare for any individual to doubt the existence of traits as essential units in the structure of personality” (Sufyan, 2004, p. 58). Narcissism is one of the most prevalent personality traits. The current era witnesses an increased focus on individuality and competition in various achievements, whether academic or professional. A narcissistic individual is characterized by a sense of grandiosity, self-importance, uniqueness, preoccupation with fantasies of unlimited success, and a need for constant admiration and attention (Abdul Khaliq, 2000).

The narcissistic personality is defined in the Cambridge Dictionary of Psychology as “an inflated self-evaluation and preoccupation with fantasies of success, power, and a sense of superiority, with a tendency to exploit others” (Al-Zahra, 2014, p. 36). Thus, a narcissistic individual is characterized by certain personality traits and moral qualities that indicate self-centeredness, where the self becomes the primary focus of attention. They judge everything based on personal gain, with a strong desire to control and dominate others, exaggerate their own virtues, and disregard the opinions, criticisms, and even the interests of others (Hussein, 2023, p. 382).

A narcissistic personality in mothers is defined as a psychological disorder that occurs in women during adulthood that is characterized by dominance, arrogance, and self-sufficiency in terms of their abilities along with a sense of entitlement. They seek to attract the attention of those around them to satisfy their egos and gain control over them, even if it involves resorting to illusion, fantasy, and the distortion of facts. This is considered a disorder in terms of behavior and imagination.

Al-Subaie (2019) described it as an addiction to the self or ego that is characterized by a constant sense of entitlement, a desire for others’ admiration, and the use of exploitation to achieve personal gains. This desire is fueled by the narcissist’s self-centeredness and sense that others were created solely to serve them and fulfill their desires, without regard for their interests or feelings. Consequently, the level of empathy that narcissistic mothers have toward others decreases, and they consider themselves more distinguished and unique than others, and he is not inclined to share them emotionally.

### Emotional balance

Avula (2020) believed that individuals can achieve emotional balance by effectively managing and regulating their emotions according to the life situations they encounter. Emotional balance has been defined as “a tendency toward compassion, tenderness, and a psychological readiness to feel specific emotional reactions and engage in certain behaviors toward a person, group, or idea” (Omar, 2008, p. 3367). The concept of emotions refers to “affective behaviors directed toward an object or situation, classified into culturally established categories or prototypes, such as fear, anger, happiness, etc.” (Scheve and Slaby, 2019, p. 2).

For their part, Ekman and Wallace (2013) defined emotional balance as “the regulation of emotions to reduce destructive emotional episodes harmful to oneself or others and increase constructive interactions to enhance understanding, communication, and human flourishing” (p. 15). Similarly, Diotaiuti et al. (2021) defined emotional balance as “the ability to control the impact of one’s mood and emotions on behavior” (p. 6). Emotionally balance is operationally defined by the researcher, Intisar, as the capacity of an individual to modulate their affective states between the poles of love and hate, avoiding extreme polarization toward either sentiment.

The importance of emotional balance lies in its being a crucial aspect of human life that is related to an individual’s adaptation to various life situations, whether social, academic, professional, or otherwise. Having emotional balance makes individuals sufficiently capable of controlling and appropriately expressing their emotions (Bhagate et al., 2015). Emotional balance helps individuals both face or alleviate stress and maintain stability while refraining from excessive emotional expression.

The significance of emotional balance lies in its fundamental role in an individual’s life because it is closely associated with the capacity for effective adaptation to diverse life situations, whether social, academic, or professional (Bhagate et al., 2015). It empowers individuals to confront stressors and regulate the expression of their emotions, while also enhancing their ability to tolerate delays in need fulfillment, cope with frustration, and engage in long-term planning (Al-Masri, 2020). Individuals possessing emotional balance are characterized by emotional maturity, self-confidence, stability in planning and emotions, and a reasoned approach to situations and facts, without marked fluctuations in mood (Pavlenko et al., 2009). Furthermore, emotional balance is considered another core personality trait and an indicator of sound mental health, existing on a continuum with its positive pole representing emotional equilibrium and its negative pole representing neuroticism, which is characterized by a tendency toward negative emotions, such as anger, anxiety, or depression, and is sometimes referred to as emotional dysregulation or instability (Jassim, 2022).

Aaron Beck’s cognitive theory focused on the role of negative thoughts in causing negative emotions and difficulty in controlling emotions. It explained that negative thoughts are distorted interpretations of reality that lead to negative emotions and unhealthy behaviors. Changing negative thoughts to realistic ones can help individuals develop their emotional balance and thereby help control their emotions and behavior (Beck, 1964).

Similarly, some behavioral specialists believe that emotions result from conflicts or problems that lead to inconsistent or inappropriate reactions. These reactions can contribute to behavioral disturbances and loss of emotional control, thus resulting in emotional imbalance. Therefore, Behavioral theory considers emotions as resulting from a lack of control over behavior, which can lead to problems for an individual (Al-Sayed Abdel, 1998). Consistent with this perspective, if we posit a university student encountering heightened academic pressure stemming from an accumulation of coursework and impending deadlines, this pressure constitutes an internal conflict and an external problem for the student. Instead of adopting healthy coping mechanisms to manage this stress (such as time management, seeking assistance, or taking breaks), the student might resort to maladaptive responses, such as excessive procrastination, social isolation, or heightened irritability toward peers and family members.

These incongruous responses may contribute to the development of behavioral disturbances (e.g., decreased academic productivity and deterioration of social relationships) and a loss of control over emotions (e.g., persistent feelings of anxiety and stress). Consequently, the student loses emotional equilibrium and becomes more susceptible to mood fluctuations and impulsive behaviors. This scenario aligns with the viewpoint of behavioral theory, which posits that negative emotions arise from a lack of control over maladaptive behaviors when confronted with stressors and problems, thereby leading to the creation of additional difficulties for the individual on both academic and personal levels.

When a narcissistic mother lacks the necessary emotional response to meet her daughter’s emotional needs, it results in emotional neglect, which is a phenomenon as real as physical deprivation. Experiencing emotional neglect in childhood can lead to painful emotional loneliness that can have long-term negative effects on daughters, especially in terms of interpersonal relationships and intimate partners (Gibson, 2015, p. 20). Successful attempts to achieve proximity and a sense of security through the attachment process are essential for establishing close relationships across various life stages. Conversely, when relationships with the mother lack trust and security, they disrupt personality development and can lead to emotional disturbances ranging from anxiety and depression to more severe personality disorders. Secure attachment reliably predicts future emotional and social competencies (Marei, 2016).

Narcissism can be considered a factor that disrupts the quality of a mother’s parenting style. More specifically, the parenting style of narcissistic mothers seems to align with the dysfunctional pattern of emotional detachment (Diotaiuti et al., 2021). Yet, it is difficult to find information about the narcissistic family system and the effects it may have on children, particularly regarding parenting. Among all of the personality traits researched and written about by renowned academics and authors, research about narcissism and the effects of narcissistic parenting is almost nonexistent, inaccurate, or scientifically unproven (McDonald, 2013).

The narcissistic personality of a mother poses an obstacle to healthy emotional upbringing for her daughters. It can lead many to struggle with managing and controlling their emotions, which can manifest in their relationships with others in terms of an extreme, misguided attachment to them. This result was supported by findings in Monk’s study (2001), which indicated that adults who grew up in narcissistic homes tend to prioritize their partners’ needs over their own, just as they prioritized their narcissistic parents’ needs over their own as a way to gain acceptance and approval.

## Materials and methods

This study, conducted among female students at King Faisal University, was designed to reveal the level of female students’ perceptions of their mothers’ narcissism and its impact on the students’ emotional balance. The primary data collection method employed in this study was an online survey conducted in Arabic, which corresponded to the native language of the female student population under investigation. Online surveys for data collection offer many advantages that align with the requirements and objectives of modern research, including convenience, efficiency, and ease of access.

A pilot study was conducted with 100 female students from King Faisal University who were not included in the primary study sample. This pilot study served to assess the suitability of the research tools for application to the sample and to determine whether the questionnaire was easy to understand, appropriate for the context, and whether the questions were unambiguous, clearly detailed, and consistently presented. Based on feedback and comments from specialists and participants in the pilot study, necessary modifications were made to the survey questionnaire, including improvements in the wording of some statements and adjustments to the sequence of questions, all of which were aimed at enhancing the clarity and overall effectiveness of the questionnaire.

The construction of the survey questionnaire involved reviewing scientific theses and research to identify scales and items related to narcissism and emotional balance. A comprehensive set of potential items for each construct was created based on the findings. The questionnaire relating to the female students’ perceptions of their mothers’ narcissism and emotional balance consisted of three sections, each serving a specific purpose. Section 1 collected basic demographic information from the participants (e.g., academic level and major area of study). Section 2 (which collected data about the female students’ perceptions of narcissism in their mothers) used a scale consisting of 45 items included within 9 dimensions, each of which contained approximately 5 items. An example of a dimension included in the survey questionnaire is “My mother sees herself as always right.” Responses to this narcissistic mother scale consisted of two potential points: one point corresponded to “sometimes,” and two points corresponded to “applies.” The term “sometimes” indicated that the item applied to the mother on certain occasions rather than consistently, as would be the case with the term “always applies”.

This scale was developed by the researcher and was based on theoretical frameworks and previous studies, such as Al-Subaie (2019), Al-Lihani (2021), Hanafi (2018), DSM-5(2013), and Hart et al. (2017). Other scales prepared in this field, including the Raskin-Hall Scale (Ali, 2018), the Kobariš, Dery, and Austin Scale (Shahada, 2018; Boldero Jennifer et al., 2015), and the narcissistic personality scale (Sufyan, 2004), were utilized to evaluate this researcher-developed scale.

Section 3 of the survey questionnaire (the emotional balance scale) aimed to explore the participants’ emotional balance using a scale that was developed by the researcher based on theoretical frameworks. Most notably, it relied on social balance theory, which is considered one of the prominent social theories that explains individuals’ behaviors and social relationships. This theory focuses on the concept of harmony among individuals in their relationships, wherein it seeks to elucidate how individuals strive to maintain equilibrium between their emotions and social evaluations.

For further detail regarding the concepts, each concept is here defined separately to explain emotional balance and its key concepts. The cognitive state is the state an individual feels concerning themselves, others, and the world around them, encompassing thoughts, feelings, and beliefs. Harmony is the state of an individual’s feeling of consistency among their thoughts, feelings, and beliefs. Dissonance refers to the state of an individual’s feeling of contradiction among their thoughts, feelings, and beliefs. Changes in cognitive state are any alteration occurring in an individual’s thoughts, feelings, or beliefs. Finally, social processes are the processes individuals employ to maintain balance in their social relationships (Festinger, 1957; Heider, 1958; Newcomb, 1959). In addition to theoretical frameworks Previous studies and metrics prepared in this field, including the emotional intelligence scale (Ghanem, 2015) and the emotional intelligence list (Jawkhb, 2008), which consists of 22 items including two dimensions. Examples of these dimensions include “I can express feelings of love.”

The potential responses for the emotional balance scale consisted of two points: one point corresponded to “sometimes” and two points corresponded to “yes.” The narcissistic mother scale and the emotional balance scale, through confirmatory factor analysis, showed acceptable fit indices, with most indices falling within their ideal ranges. This analysis indicated that both scales demonstrated high validity, thereby permitting their application to the main study sample with substantial reliability, as illustrated in the subsequent tables and figures.

**Figure 1:**
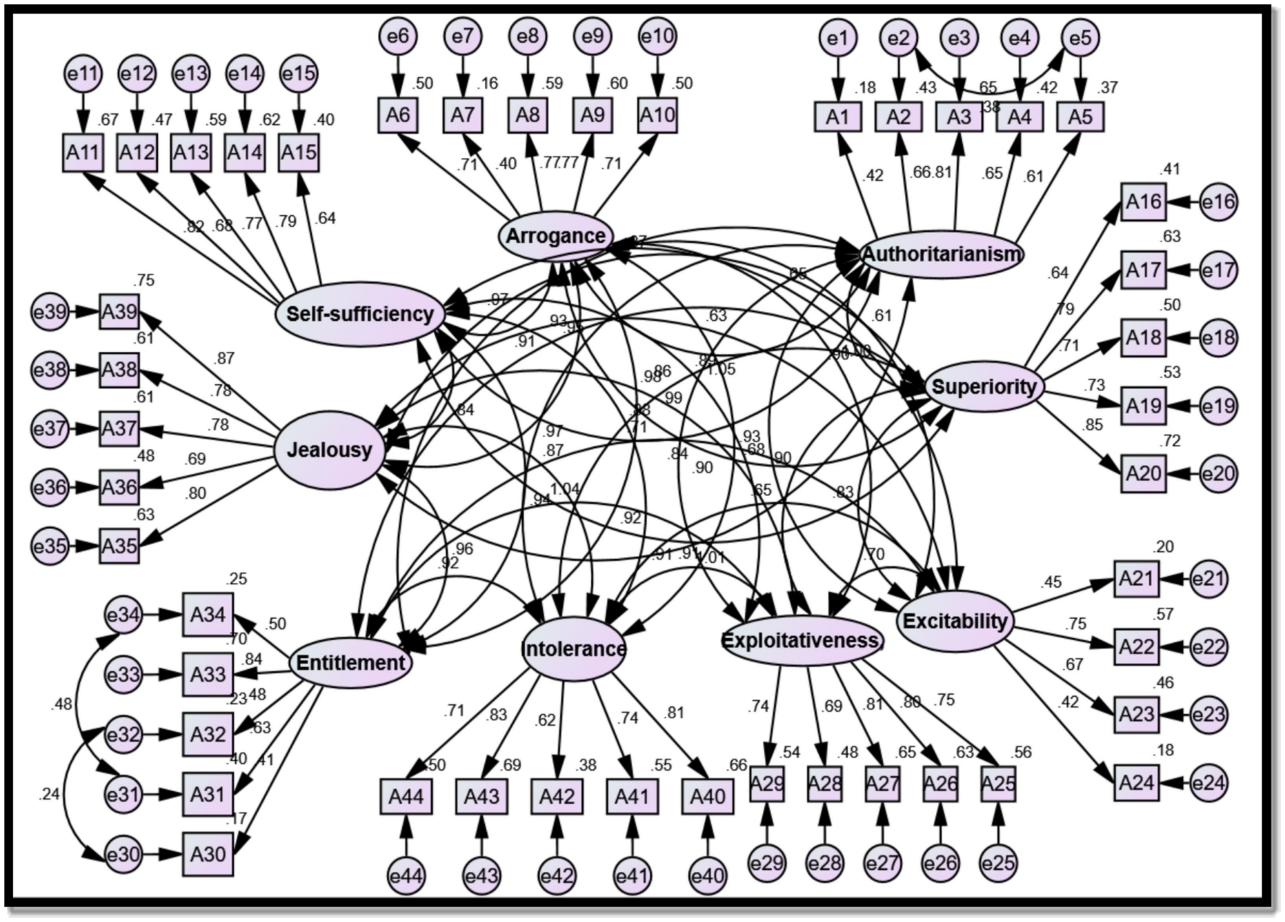
Confirmatory factor analysis of the narcissistic mother scale.

The validity of the current study’s scale assessing female students’ perceptions of their mothers’ narcissistic personalities was examined through confirmatory factor analysis (CFA) using AMOS v24 after administering the scale to the pilot study participants (*n* = 100). It was hypothesized that the latent factors of the scale would organize into nine factors: factor 1 (dominance), factor 2 (arrogance), factor 3 (self-sufficiency), factor 4 (superiority), factor 5 (excitability), factor 6 (exploitativeness), factor 7 (entitlement), factor 8 (jealousy), and factor 9 (intolerance). These nine factors comprised a total of 44 observed variables. Specifically, all factors are distributed by 5 viewing factors, except for the excitability factor, which is distributed by 4 viewing factors. Table 1 and Figure 1 illustrate the CFA of the scale assessing female students’ perceptions of their mothers’ narcissism.

**Table 1:** Fit indices for the confirmatory factor analysis model of the narcissistic mother scale (*n* = 100).

Fit indices	Value	Ideal range
Chi-square/df ratio (*X*^2^/df)	2.115	0–3
Goodness of Fit Index (GFI)	0.924	0.90–1
Incremental Fit Index (IFI)	0.903	
Relative Fit Index (RFI)	0.910	
Normed Fit Index (NFI)	0.897	
Tucker-Lewis Index (TLI)	0.931	
Root Mean Square Error of Approximation (RMSEA)	0.072	0–0.08
Expected Cross-Validation Index (ECVI)	20.889	Lower than saturated model
ECVI for Saturated Model	21.892	
Akaike Information Criterion (AIC)	2068.00	Lower than saturated model

**Figure 2:**
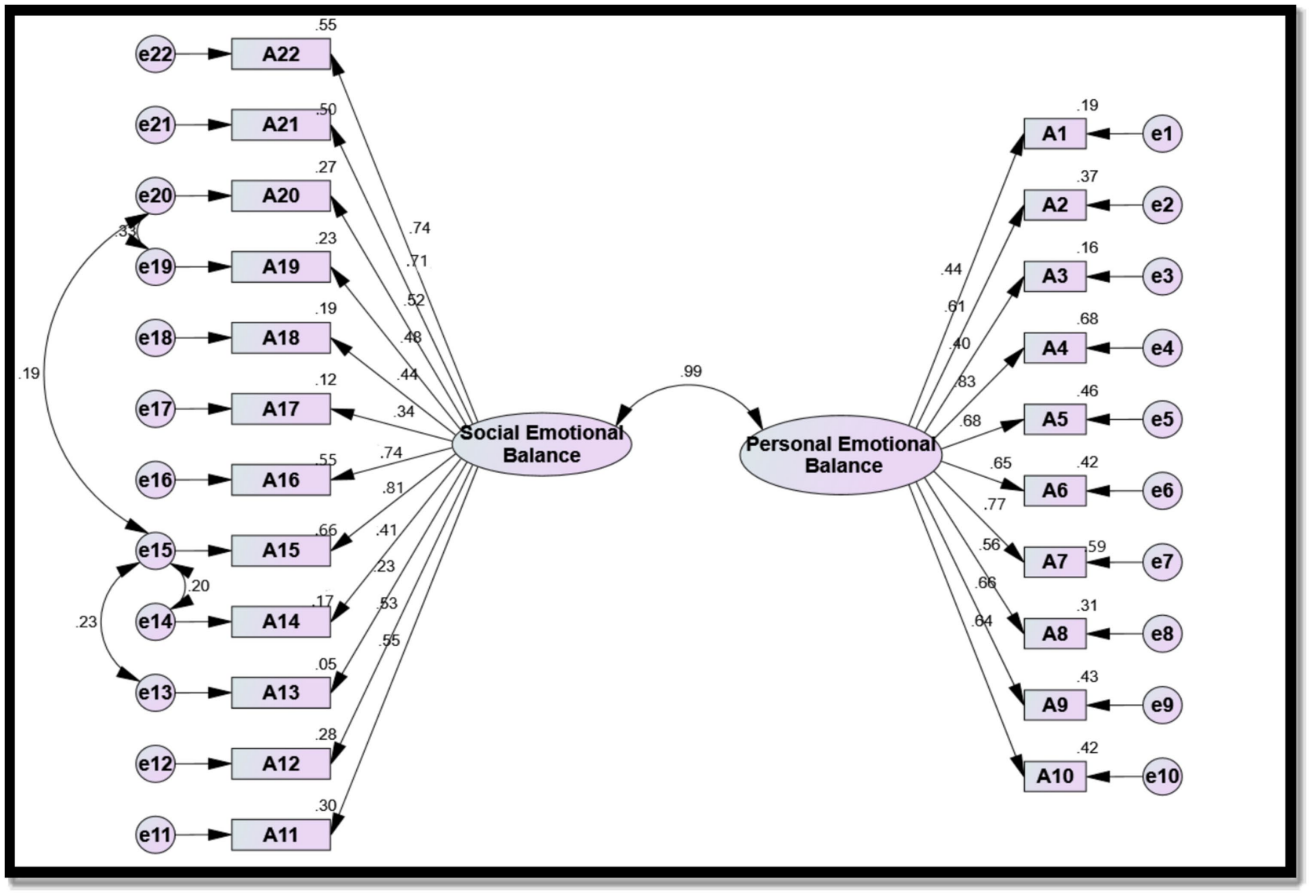
Confirmatory factor analysis of the emotional balance scale.

The validity of the emotional balance scale in the current study was examined through CFA using AMOS v24 after administering the scale to the pilot study participants (*n* = 100). It was hypothesized that the latent factors of the scale would organize into two factors: factor 1 (personal emotional balance) and factor 2 (social emotional balance). These two factors comprised a total of 22 observed variables. Specifically, factor 1 consisted of 10 observed variables, and factor 2 comprised 12 observed variables. Table 2 and Figure 2 illustrate the CFA of the emotional balance scale.

**Table 2:** Fit indices for the confirmatory factor analysis model of the emotional balance scale (*n* = 100).

Fit indices	Value	Ideal range
Chi-square/df ratio (X^2^/df)	2.240	0–3
Goodness of Fit Index (GFI)	0.920	0.90–1
Incremental Fit Index (IFI)	0.904	
Relative Fit Index (RFI)	0.932	
Normed Fit Index (NFI)	0.900	
Tucker-Lewis Index (TLI)	0.914	
Root Mean Square Error of Approximation (RMSEA)	0.067	0–0.08
Expected Cross-Validation Index (ECVI)	5.556	Lower than saturated model
ECVI for Saturated Model	6.051	
Akaike Information Criterion (AIC)	550.00	Lower than saturated model
AIC for Saturated Model	599.045	

A strong internal consistency was observed for the narcissistic mother scale (*α* = 0.891) and the emotional balance scale (*α* = 0.803).

### Evaluation of the measurement scales

After the data collection phase, the psychometric properties were evaluated for validity and reliability using the AMOS v24 program and included inter-rater reliability and CFA validity after applying the scale to the pilot study participants (*n* = 100). The results shown in Tables 3, 4 illustrate the positive psychometric properties.

**Table 3:** Reliability coefficients for the dimensions of the narcissistic mother scale and the emotional balance scale using Cronbach’s alpha for the total score and sub-dimensions of the scale (*n* = 100).

No.	Narcissism dimensions	Cronbach’s alpha	Emotional balance dimensions	Cronbach’s alpha
1	Dominance	0.809	Personal emotional balance	0.654
2	Arrogance	0.807	Social emotional balance	0.725
3	Self-Sufficiency	0.853		
4	Superiority	0.860		
5	Excitability	0.767		
6	Exploitativeness	0.867		
7	Entitlement	0.734		
8	Jealousy	0.891		
9	Intolerance	0.855		
Total scale		0.970	Total scale	0.803

**Table 4:** Reliability coefficients for the dimensions of the emotional balance scale using Cronbach’s alpha (*n* = 100).

No.	Dimensions	Cronbach’s Alpha
1	Personal emotional balance	0.654
2	Social emotional balance	0.725
Total scale		0.803

### Sampling methodology and data collection procedures

The current study relied on the use of rating scales as a standard tool for assessing personality. Rating scales in this field are tools built on a specific trait, whether social, moral, or emotional, to measure an individual’s mental health or maladjustment or to evaluate positive personality traits or symptoms of mental illness. Such rating scales can be answered by individuals themselves or by peers at school, university, or work, or by a psychologist who observes an individual for a period of time (Al-Khaliq, 1996).

This study focused on university students because they are particularly susceptible to emotional fluctuations, given the various situations they face at university or at home. They are also subject to surrounding influences that include factors that place them in the midst of various problems and challenges. These circumstances can stimulate their emotions and cause behavioral disturbances that can result in negative effects on their lives (Al-Masri, 2020).

The study targeted female students at King Faisal University as a model representing the Saudi environment. After obtaining ethical approval from the Scientific Research Ethics Committee at King Faisal University for the research tools and procedures, the study scales (the narcissistic mother scale and the emotional balance scale) were prepared by the researcher and presented to a number of arbitrators for review. The scales were finalized in the form of an electronic questionnaire distributed through the WhatsApp social media application to female students at King Faisal University. Challenges were encountered in terms of persuading the female students to reply, so the link was shared multiple times to encourage responses. Furthermore, a paper-based questionnaire was directly distributed to female students at the university, We received 245 paper responses to increase the number of responses. The final sample size was 416 female students aged between 18 and 24 years, and data collection took approximately 3 months from the end of January to April 2023. The researcher acquired the study sample data through WhatsApp groups to which some female students of King Faisal University belong.

### Data analysis

The data pertaining to this study were analyzed using the SPSS software and employing the following statistical methods: Pearson’s correlation coefficient to ascertain the internal consistency of the two scales, which revealed that all correlation coefficients between each item’s score and the total score were statistically significant at a significance level of (0.01), indicating a high degree of internal consistency for both scales. Calculation of means and standard deviations was conducted for the scores of the sample members across the dimensions. Calculation of the hypothetical mean of the scales was completed to examine the validity of the study’s research questions. Simple linear regression analysis was employed, revealing a statistically significant predictive relationship (*β* = 0.144-, *p* < 0.01) between perceived maternal narcissistic personality traits and emotional equilibrium. Finally, CFA was used to verify the validity of the two scales. Both scales exhibited a high degree of validity, thus permitting their application to the primary study sample with a high level of reliability.

## Results

### The demographic characteristics of the respondents

The study collected a total of 416 valid responses from undergraduate female students at King Faisal University enrolled during the academic year 1444/1445 AH. The majority of respondents were from the College of Agricultural Sciences, comprising 53.37%, followed by Business Administration at 25.24%, Natural Sciences at 10.34%, and the College of Arts at 9.38%. The remaining 1.68% were from other academic majors. Regarding the participants’ academic levels, defined as the hierarchical classification of students based on their progress in a specific academic program and typically reflecting the number of successfully completed credit hours, 61.54% were in levels 1–3, followed by levels 4–6 at 25.48%. The higher academic levels, 7 and 8, constituted the smallest proportion at 12.98%. These percentages represented the response rate of students from each specialization relative to the total sample of 416. The study did not gather information concerning the mothers’ ages, the number of children in the family, or the participants’ birth order within their families, but rather focused solely on undergraduate female students at King Faisal University across various academic specializations and all their respective academic levels.

### Results of the first research question

“What is the level of female students at King Faisal University’s perception of their mothers’ narcissistic personality?”

To test the validity of this question, the means and standard deviations of the respondents’ scores on the dimensions of their perceptions of their mothers’ narcissism, or the total score, were calculated, as well as the hypothetical mean for the scale. The hypothetical mean for the scale was calculated by summing the response options in the scale, dividing them by their number, and then multiplying the result by the number of items. Therefore, the weights of the alternatives were 2, 1, and 0, their sum was 3, and their number was 2, so the average weight of the alternatives was 1. When multiplied by the number of items for each dimension (1 × 5), eight of the nine dimensions had a predetermined mean of 5 (comprising five items each), whereas the fifth dimension, consisting of four items, had a mean of 4. Consequently, the total scale’s baseline mean equaled 44 (1 × 44 items).

A one-sample t test was used to verify the significance of the differences between the hypothetical mean and the arithmetic mean of the respondents’ scores on the narcissistic mother scale. When the t value is statistically significant, it indicates the presence of differences between the two means, and the differences are directed in favor of the higher mean. If the differences are in favor of the higher mean, it indicates a high level, while if the differences are in favor of the lower mean, it indicates a low level. The nonsignificance of the t value indicates no differences between the two means, indicating an average level. Table 5 shows the results.

**Table 5:** Results of the one-sample t-test for differences between the hypothetical mean and the arithmetic mean in the narcissistic mother scale (*n* = 416).

Dimensions	Arithmetic mean	Standard deviation	Hypothetical mean	*t* value	Significance level	Level
Dominance	2.42	2.47	5	−21.28	0.01	Low
Arrogance	1.67	2.10	5	−32.79	0.01	Low
Self-Sufficiency	3.16	2.74	5	−13.67	0.01	Low
Superiority	3.19	2.82	5	−13.10	0.01	Low
Excitability	4.10	2.18	4	0.72	Not significant	Average
Exploitativeness	2.10	2.47	5	−24.37	0.01	Low
Entitlement	2.50	2.30	5	−21.13	0.01	Low
Jealousy	1.61	2.10	5	−33.36	0.01	Low
Intolerance	2.52	2.35	5	−21.53	0.01	Low
Total score	23.20	16.71	44	−25.39	0.01	Low

The results in Table 5 show that the t values were statistically significant, indicating the presence of statistically significant differences at the 0.01 level between the hypothetical mean and the arithmetic mean of the respondents’ scores on the narcissistic mother scale, in favor of the hypothetical mean, except for the “excitability” dimension. This result indicated that the female students had a low level of perception of narcissism in their mothers except for the “excitability” dimension, which was average.

### Results of the second research question

“What is the level of emotional balance among a sample of female students at King Faisal University whose mothers are narcissistic?”

To test the validity of this question, the means and standard deviations of the respondents’ scores on the dimensions of the emotional balance scale, or the total score, were calculated, as well as the hypothetical mean for the scale. The hypothetical mean for the scale was calculated by summing the response options in the scale, dividing them by their number, and then multiplying the result by the number of items. Therefore, the weights of the alternatives were 2, 1, and 0, their sum was 3, and their number was 2, so the average weight of the alternatives was 1. When multiplied by the number of items for each dimension of the scale, for the first dimension (1 × 10), the hypothetical mean was 10, and for the second dimension it was 1 × 12 = 12. The hypothetical mean for the total scale score was 1 × 22 = 22. Table 6 shows the results of these calculations.

**Table 6:** Results of the one-sample *t*-test for differences between the hypothetical mean and the arithmetic mean in emotional balance (*n* = 416).

Dimensions	Arithmetic mean	Standard deviation	Hypothetical mean	*t*-value	Significance level	Level
Personal emotional balance	10.87	3.45	10	5.10	0.01	High
Social emotional balance	11.29	3.91	12	3.72	0.01	High
Total score	22.16	6.03	22	0.54	Not significant	Average

The results in Table 6 show that the t values were statistically significant at the 0.01 level. The results indicated statistically significant discrepancies between the hypothetical mean and arithmetic mean scores in both the personal equilibrium [*t* (98) = 3.21, *p* < 0.01] and social equilibrium [*t* (98) = 2.89, *p* < 0.05] dimensions, which suggested elevated proficiency levels among female students in these specific domains. However, no significant difference emerged in the composite emotional equilibrium score [*t* (98) = 1.12, *p* > 0.05], which indicated average overall emotional balance.

### Verification of the first hypothesis

This hypothesis stated, “There is a statistically significant negative correlation between the perceptions of female students at King Faisal University of narcissism in their mothers and their scores on the emotional balance scale”.

To test the validity of this hypothesis, Pearson’s correlation coefficient was calculated between the raw scores of the female student respondents in terms of their perceptions of narcissism in their mothers and their emotional equilibrium scores. Table 7 presents these results in detail.

**Table 7:** Correlation coefficients between the respondents’ scores on the narcissistic mother scale (total score - sub-dimensions) and their scores on the emotional balance scale (*n* = 416).

Dimensions	Emotional balance	Personal emotional balance	Social emotional balance	Total score
Perception of mothers’ narcissism	Dominance	−0.393**	−0.151**	−0.325**
	Arrogance	−0.385**	−0.178**	−0.338**
	Self-Sufficiency	−0.346**	−0.207**	−0.334**
	Superiority	−0.307**	−0.165**	−0.284**
	Excitability	−0.10*	−0.132**	−0.142**
	Exploitativeness	−0.422**	−0.187**	−0.365**
	Entitlement	−0.380**	−0.169**	−0.329**
	Jealousy	−0.313**	−0.151**	−0.278**
	Intolerance	−0.430**	−0.207**	−0.382**
	Total score	−0.441**	−0.222**	−0.339**

*p < 0.05; **p < 0.01.

The results in Table 7 indicate the following observations.

- There were statistically significant negative correlations at the 0.01 level between the dimensions of the respondents’ perceptions of narcissism in their mothers and the total score and the personal emotional balance dimension, with correlation coefficients ranging between −0.10 and −0.441.
- There were statistically significant negative correlations at the 0.01 level between the dimensions of the respondents’ perceptions of narcissism in their mothers and the total score and the social emotional balance dimension, with correlation coefficients ranging between −0.132 and −0.222.
- There was a statistically significant negative correlation at the 0.01 level between the total score of the respondents’ perceptions of narcissism in their mothers and the total score of emotional balance. The correlation coefficient reached a value of −0.339 (*p* < 0.05), indicating a statistically significant moderate inverse relationship.

### Verification of the second hypothesis

This hypothesis stated, “The presence of maternal narcissism significantly contributes to the prediction of emotional balance in a sample of university female students”.

To verify the validity of this hypothesis, the researcher took two steps:

*First step*: Verification of the contribution of the total score of the perceptions of narcissism in mothers in predicting the emotional balance of female students at King Faisal University. Simple linear regression analysis was conducted with perceived maternal narcissistic personality traits as the independent variable and emotional equilibrium as the dependent variable (y). Table 8 shows the results.

**Table 8:** Analysis of variance results for the regression model between the perceptions of narcissism in mothers (total score) and emotional balance.

Model	Source	Sum of squares	Df	Mean square	*F*-value	Significance level
1	Regression	2405.679	1	2405.679	78.237	0.000
	Residual	12729.850	414	30.748		
	Total	15135.529	415			

The results in Table 9 show that there was a statistically significant regression coefficient at the 0.01 level for perceptions of narcissism in mothers in terms of predicting emotional balance, with a beta value of −0.144, and an *R*^2^ value of 0.159. Therefore, the total score of the perceptions of narcissism in mothers explained 15.9% of the variance in emotional balance. The predictive equation can be written as follows: Emotional Balance = 25.501–0.144 × Perceptions of Narcissism in Mothers.

**Table 9:** Linear regression analysis values for predicting emotional balance through the total score of the perceptions of narcissism in mothers.

Variable	Constant	Standardized regression coefficient (Beta)	*t* value	Correlation coefficient (R)	Coefficient of determination (*R*^2^)
Perception of mothers’ narcissism	25.501	−0.144	−8.845	0.399	0.159

*p* < 0.01.

*Second step*: Verification of the contribution of the dimensions of the perceptions of narcissism in mothers in predicting the emotional balance of female students at King Faisal University was conducted to determine which dimension of the perceptions of narcissism in mothers was the most predictive of emotional balance. Stepwise multiple regression analysis was used, which considered emotional balance as the dependent variable and the dimensions of the perceptions of narcissism in mothers as independent variables. Table 10 shows the results of these calculations (Table 11).

**Table 10:** Analysis of variance results for the regression model between the variables.

Model	Source	Sum of squares	Df	Mean square	*F*-value	Significance level
1	Regression	2211.622	1	2211.622	70.848	0.000
2	Residual	12923.867	414	31.217		
	Total	15135.529	415			
	Regression	2564.439	1	2564.439	42.125	0.000
	Residual	12571.090	414	30.438		
	Total	15135.529	415			

**Table 11:** Multiple regression analysis values for predicting emotional balance from the dimensions of the perceptions of mothers’ narcissism.

Variables	Constant	Standard error	Standardized regression coefficient (Beta)	*t* value	Correlation coefficient (R)	Coefficient of determination (*R*^2^)
Constant	24.639	0.402		−8.417**	0.382	0.146
Intolerance	−0.984	0.117	−0.382	−12.855**		
Constant	24.801	0.400			0.456	0.208
Intolerance	−0.647	0.152	−0.251	−4.256**		
Exploitativeness	−0.492	0.145	−0.201	−3.404**		

**p < 0.01.

The results in Table 10 indicate that the *F*-values for the stepwise regression models were significant at the 0.001 level, and the first regression model indicated that “intolerance” was the most significant variable in predicting emotional balance, with a coefficient of determination (*R*^2^) of 0.146. This meant that approximately 14.6% of the variance in emotional balance could be explained by this variable. The second model indicated that “exploitativeness” was the second most significant variable in predicting emotional balance, with a coefficient of determination (*R*^2^) of 0.208. This meant that approximately 20.8% of the variance in emotional balance could be explained by these two variables.

Based on the results of the two-step regression analysis, the predictive equation could be formulated to include the independent variables that made a statistically significant contribution to predicting emotional balance, as follows.


$$\begin{array}{l} \text{Emotional Balance}=24.801–0.152\\ \times \text{Intolerance}–0.145\times \text{Exploitativeness} \end{array}$$

## Discussion

The main objective of this study was to reveal the level of female students’ perceptions of their mothers’ narcissistic personalities and their emotional balance to verify the nature of the relationship between the perception of narcissism in their mothers by daughters and its relationship with the daughters’ emotional balance. The study hypothesized that daughters’ perceptions of their mothers’ narcissism would negatively affect their emotional balance and that the mothers’ narcissism would contribute to predicting the daughters’ emotional balance. First, the relationship between the two variables was calculated to determine the correlation between them. The study revealed the interaction between the daughters’ perceptions of their mothers’ narcissism and their emotional balance as daughters of narcissistic mothers.

The correlation results in the current study indicated an inverse relationship between the perception of the mothers’ narcissism and the emotional balance of the students. This meant that the higher the students’ perceptions of their mothers’ narcissism, the lower their scores were on the emotional balance scale. Conversely, the lower their perceptions of their mothers’ narcissism, the higher their scores were on the emotional balance scale. This relationship can be explained by the fact that being raised by a narcissistic mother can negatively affect the development of emotional balance.

These findings align with the study by Al-Harbi (2016), which demonstrated a statistically significant negative correlation (*r* = −0.42, *p* < 0.01) between perceived maternal narcissistic personality traits and emotional equilibrium among university students. This inverse relationship indicates that female students reporting higher levels of maternal narcissism perception exhibited significantly lower emotional balance (*M* = 2.31, SD = 0.56 vs. *M* = 3.45, SD = 0.62), and vice versa.

Similarly, Al-Atibi’s (2017) research revealed a significant negative association (*β* = − 0.38, *p* < 0.05) between narcissistic parenting styles and adolescent emotional regulation, thus suggesting that adolescents raised by narcissistic mothers scored markedly lower on emotional stability scales [95% CI (−0.87, −0.32)] compared to their peers.

These results provide strong support for the idea that a narcissistic mother has a harmful effect on the emotional balance of her daughters. This is supported by attachment theory, which suggests that humans are born with a strong need to form emotional bonds with primary caregivers, especially mothers. If a daughter does not receive sufficient emotional care from her mother, she is likely to face difficulties in developing her emotional and social skills, which may lead to lower levels of emotional balance (Bowlby, 1982). Similarly, self-theory suggests that humans develop a mental image of themselves consisting of their thoughts, feelings, and beliefs about those around them. If a daughter is raised by a narcissistic mother, she is likely to develop a negative self-image, which may lead to lower levels of emotional balance (Markus, 1977). Alice Miller, a psychologist, physician, and researcher in developmental psychology and childhood trauma effects and the originator of toxic parenting theory referred to this in her study (Monk, 2001) by stating that children resulting from one or two narcissistic parents may thrive intellectually, but their emotional lives remain empty.

This relationship may be explained by psychological defense mechanisms. Students raised in narcissistic families may resort to defense mechanisms that affect their emotional balance, such as denial or displacement. This is consistent with Donaldson—a developmental psychologist specializing in child cognitive growth and the originator of the social context of cognition theory—and Briseman (a child and adolescent psychiatrist specializing in narcissistic parenting dynamics), whose seminal contributions included groundbreaking research on the multigenerational transmission of narcissistic traits and their developmental consequences in offspring interpretation of their social theory, which suggested that children in narcissistic families may not be taught to set boundaries in their interactions with their parents. As adults, they may prioritize others’ needs over their own and rarely express what they want, how they feel, or what they need. They are often completely unaware that they have needs apart from what others want from them because, in narcissistic family systems, children are not allowed to have feelings, thoughts, or expressions that do not reflect a positive image of their parents. Therefore, they learn to hide their true feelings (McDonald, 2013). Additionally, Monk (2001) noted that adults who grew up in narcissistic families feel the need to be physically and emotionally available to anyone at any time.

This inverse relationship between the perception of the mothers’ narcissism and the emotional balance of the students may also be explained by negative expectations. Students raised in narcissistic families may have negative expectations about relationships, which may affect their emotional balance. Additionally, it may be explained by emotional difficulties, because students raised in narcissistic families may suffer from emotional difficulties, such as low self-esteem or poor social skills, which affect their emotional balance. This is supported by Bach’s (2014) study, for instance, affirmed that individuals raised by narcissistic parents may experience emotional disorders and encounter difficulties in comprehending the nature of potential future relationships. Similarly, Maatta et al. (2020) corroborated the findings of the current study, which indicates the challenges faced by offspring, specifically a sample of female university students, in achieving emotional equilibrium as a result of being raised by narcissistic mothers.

This result indicated that individuals raised by mothers exhibiting narcissistic traits encounter significant difficulties in achieving a state of stability and equilibrium in their emotions and reactions. This difficulty is not limited to childhood and adolescence but may persist into adulthood.

The results of this study have important theoretical implications in the field of family counseling and the future emotional lives of daughters. They showed that a mother’s narcissistic personality can predict a troubled future for the emotional balance of daughters who are raised in an environment that lacks emotions, empathy, and tolerance. The results are consistent with the study by Monk (2001), which found that adults who came from narcissistic families indeed suffered from basic trust and intimacy issues that affected their ability to establish and maintain satisfying emotional relationships. Similarly, Yorkovich, a researcher in educational psychology and psychometrics whose scholarly contributions have advanced the field of pedagogical assessment through empirically validated measurement methodologies, noted in the same study that the disturbed personalities of parents may lead to the children having a lifelong inability to express negative emotions, which may lead to unsatisfactory and unfulfilling jobs, friendships, and emotional bonds. This was also indicated by Dr. Mohammed Al-Obaidi, as quoted by Al-Obaidi (2006), p. 45 who stated that verbal abuse, feelings of guilt, and hatred hinder the social adjustment of children.

Sullivan’s social relations theory emphasizes the significant influence of parental upbringing on child development. Sullivan (Al-Safi, 2018, pp. 112–115) posits that individuals enter into social relationships from birth, and that interactions with parents during childhood socialization constitute acceptable behavior, determining the child’s sense of security or insecurity based on maternal behaviors. Consequently, the child becomes sensitive to others’ attitudes toward the self, and this sensitivity develops and is reinforced through the early integration of empathy. Harry Stack Sullivan, a pioneer in social psychology and the interpersonal theory of psychoanalysis, described empathy in its early stages as “emotional contagion,” explaining that infants, despite lacking a sophisticated cognitive capacity to comprehend others’ complex emotions, possess a fundamental mechanism for perceiving the affective states of close individuals, particularly the primary caregiver, through nonverbal channels such as facial expressions, tone of voice, and body language, thereby eliciting congruent emotional responses. In essence, children experience the emotions expressed by those around them and begin to respond reflexively, with this initial “emotional contagion” forming the basis for the more complex development of empathy in later developmental stages (Sullivan, 1953, pp. 45–47).

## Conclusion

This study integrated two variables (maternal narcissism and emotional balance) to determine the relationship between them by measuring the level of maternal narcissistic traits according to their daughters’ perceptions of the presence of these traits. Female students at King Faisal University were selected as the sample for this study, which yielded the following results.

The level of the students’ perceptions of maternal narcissistic personalities was low, with the exception of the excitability dimension, which was moderate. The overall level of emotional balance was moderate. Statistically significant negative correlations were found at the 0.01 level between the dimensions of perceived maternal narcissistic personality and the total score and the personal emotional balance dimension. Statistically significant negative correlations were found at the 0.01 level between the dimensions of perceived maternal narcissistic personality and the total score and the social emotional balance dimension.

In addition, a statistically significant negative correlation was found at the 0.01 level between the total score of perceived maternal narcissistic personality and the total score of emotional balance. Intolerance emerged as the most significant predictor of emotional balance, while the exploitativeness variable was the second most significant predictor of emotional balance.

The researcher proposes the following topics for future research.

- An intervention program to mitigate the impact of maternal narcissism on daughters in the university stage.
- A study of the relationship between maternal narcissistic personality and certain psychological variables among their offspring in the university stage.
- A comparative study of the level of emotional balance between daughters of narcissistic mothers and their typical peers in the university stage.

## Data Availability

The raw data supporting the conclusions of this article will be made available by the authors, without undue reservation.
